# Multimodal eye-tracking reveals impaired inhibitory control and reduced preparatory pupil dilation in essential tremor

**DOI:** 10.1093/braincomms/fcag389

**Published:** 2026-10-15

**Authors:** Chien-Tai Hong, Jui-Tai Chen, Audrey Yong, Ta-Liang Chen, Kevin S Xu, Chin-An Wang

**Affiliations:** Department of Neurology, School of Medicine, College of Medicine, Taipei Medical University, Taipei 110, Taiwan; Department of Neurology, Shuang Ho Hospital, Taipei Medical University, Taipei 235, Taiwan; Department of Anesthesiology, School of Medicine, College of Medicine, Taipei Medical University, Taipei 110, Taiwan; Department of Anesthesiology, Shuang Ho Hospital, Taipei Medical University, Taipei 235, Taiwan; Eye-Tracking Laboratory, Shuang Ho Hospital, Taipei Medical University, Taipei 235, Taiwan; College of Medicine, Chang Gung University, Taoyuan 333, Taiwan; Department of Anesthesiology, School of Medicine, College of Medicine, Taipei Medical University, Taipei 110, Taiwan; Health Policy Research Center, Taipei Medical University Hospital, Taipei 110, Taiwan; Department of Anesthesiology, Wang Fang Hospital, Taipei Medical University, Taipei 116, Taiwan; Department of Computer and Data Sciences, Case Western Reserve University, Cleveland, OH 44106, USA; Department of Anesthesiology, School of Medicine, College of Medicine, Taipei Medical University, Taipei 110, Taiwan; Department of Anesthesiology, Shuang Ho Hospital, Taipei Medical University, Taipei 235, Taiwan; Eye-Tracking Laboratory, Shuang Ho Hospital, Taipei Medical University, Taipei 235, Taiwan; Department of Education and Humanities in Medicine, School of Medicine, College of Medicine, Taipei Medical University, Taipei 110, Taiwan; Ph.D. Program in Medical Neuroscience, College of Medical Science and Technology, Taipei Medical University, Taipei 235, Taiwan

**Keywords:** essential tremor, oculomotor control, pupillometry, autonomic function, inhibitory control

## Abstract

Essential tremor (ET) is increasingly recognized as a disorder involving cerebello-thalamo-cortical and autonomic processes beyond tremor itself. Oculomotor, pupillary and blink signals provide complementary physiological readouts of these distributed systems, yet they have largely been studied in isolation. Here, we used video-based eye tracking during an interleaved pro- and anti-saccade task to characterize oculomotor performance, pupil dynamics, blink behaviour and trial-by-trial cross-domain coupling in 45 ET patients and 46 controls. ET showed impaired inhibitory oculomotor control, reflected by increased direction errors, prolonged anti-saccade reaction times, extended voluntary override time and reduced generation of express saccades. Preparatory pupil dilation was attenuated and constriction timing delayed despite a preserved tonic baseline. In contrast, blink dynamics were largely preserved. Trial-by-trial pupil–saccade coupling was also largely preserved, indicating maintained cross-domain coordination despite reduced preparatory autonomic modulation. Exploratory machine-learning analyses suggested that combined pupil–saccade features may capture aspects of clinical heterogeneity, particularly early onset status, although these findings did not survive permutation testing. Together, these results indicate that ET is associated with selective impairment of inhibitory oculomotor control and preparatory autonomic responses, with preserved cross-domain coordination. Multimodal eye tracking may therefore provide a scalable, non-invasive framework for characterizing the heterogeneous physiological expression of ET.

## Teaser

Hong *et al*. report that essential tremor is associated with impaired inhibitory eye-movement control and reduced preparatory pupil dilation. Blink dynamics and cross-domain coordination are largely preserved, suggesting selective rather than global disruption of eye-movement and pupil-linked arousal processes.

## Graphical Abstract

**Graphical Abstract:**
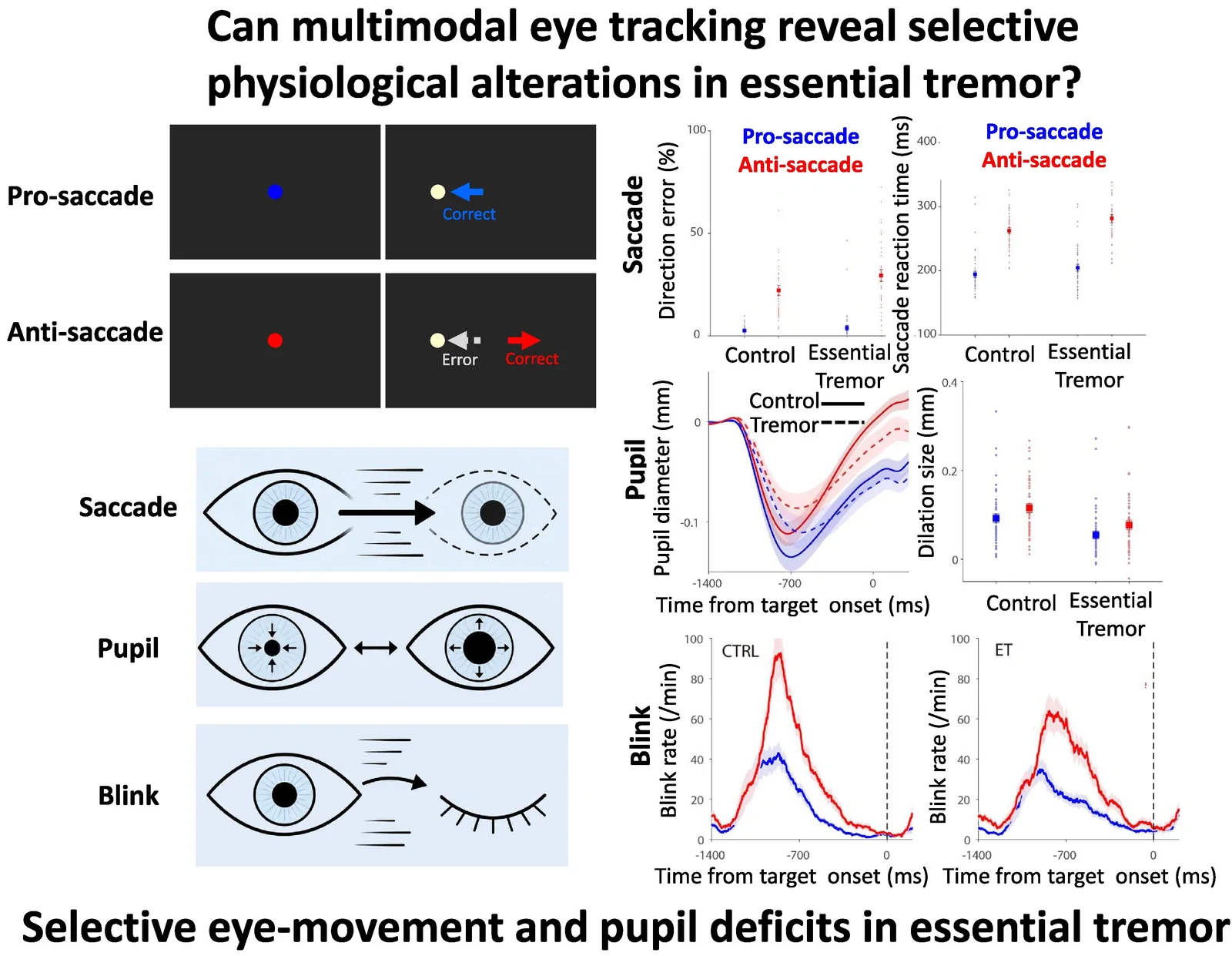

## Introduction

Essential tremor (ET) is among the most prevalent movement disorders, affecting ∼1% of the general population and more than 4% of individuals older than 65 years.^1,2^ Although traditionally defined by isolated upper-limb action tremor, current clinical frameworks distinguish ‘isolated ET’ from ‘ET-plus’, reflecting involvement beyond the core motor syndrome.^3^ Converging evidence from neuropathology and neuroimaging implicates dysfunction within cerebello-thalamo-cortical circuits, with additional abnormalities extending to sensorimotor, cognitive and autonomic domains.^4-7^ Consistent with this broader profile, ET is associated with a substantial non-motor burden,^8-11^ including impairments in inhibitory control, attentional set maintenance and working memory,^9,12^ and in some cases features resembling cerebellar cognitive affective syndrome.^13,14^ As these functions are critical for suppressing prepotent responses, executive-control paradigms provide a targeted approach to probing vulnerable circuits in ET.

Video-based eye tracking provides a non-invasive approach to assessing distributed physiological processes in ET. Within a single session, saccadic behaviour, pupil dynamics and blink kinematics can be recorded simultaneously, each indexing partially overlapping neural systems. Oculomotor control relies on frontoparietal, basal ganglia, superior colliculus (SC) and cerebellar circuits that overlap with those implicated in ET.^15,16^ Pupil signals provide an indirect index of autonomic and neuromodulatory state: tonic diameter has been associated with baseline arousal, whereas task-evoked dilation has been linked to phasic pupil-linked arousal responses, including contributions from locus coeruleus–norepinephrine (LC–NE)-related systems.^17-19^ Blink kinematics reflect integrated activity across brainstem, cerebellar and basal ganglia networks.^20,21^ Together, these measures provide complementary indices of oculomotor and autonomic function that can be assessed within a single experimental paradigm.

The interleaved pro- and anti-saccade task provides a useful framework for probing automatic visuomotor orienting and voluntary control.^22,23^ Pro-saccades involve reflexive gaze shifts towards a peripheral target, whereas anti-saccades require suppression of this prepotent response and generation of a saccade in the opposite direction, engaging frontoparietal and cerebellar circuits in addition to core sensorimotor pathways.^16,24,25^ Importantly, because pro- and anti-saccade trials are randomly interleaved, performance also depends on attentional monitoring, working-memory maintenance of task rules and flexible response selection. Thus, deficits in this paradigm are best interpreted as reflecting inhibitory control under executive task demands, rather than a pure measure of oculomotor inhibition. Prior studies using oculomotor paradigms have reported impaired saccadic performance in ET,^26-28^ and increased anti-saccade error rates have been linked to executive dysfunction within cerebello-thalamo-cortical circuitry.^29^ The interleaved design further enables quantification of voluntary override time (VOT), indexing the efficiency of suppressing prepotent responses, and express saccades, indexing rapid sensorimotor transformation via retinotectal pathways. These measures therefore provide a sensitive experimental framework for assessing inhibitory control and sensorimotor processing under executive-control demands.

Beyond oculomotor measures, pupil and blink dynamics provide complementary indices of autonomic–noradrenergic and brainstem–cerebellar function. Despite evidence for broader physiological involvement in ET, these signals have largely been studied in isolation. It therefore remains unclear whether abnormalities across domains reflect parallel impairments or changes in their coordination. Prior work suggests that task-evoked pupil dilation reflects phasic engagement of LC–NE systems, whereas baseline pupil size indexes tonic arousal, raising the possibility that these components may be differentially affected in ET. To address this, we used video-based eye tracking during an interleaved pro- and anti-saccade task to simultaneously quantify oculomotor performance, pupil dynamics and blink behaviour. We hypothesized that ET would show impaired inhibitory control and altered pupil dynamics, with potential differences between baseline and task-evoked pupil responses. We further examined whether trial-by-trial relationships between oculomotor, pupillary and blink measures were altered, to assess cross-domain coordination in ET.

## Materials and methods

### Experimental setup

All procedures were approved by the Institutional Review Board of Taipei Medical University and conformed to the Declaration of Helsinki. Participants provided written informed consent and received compensation. Fifty patients with ET and 50 healthy older adults were recruited; after excluding participants with >50% missing trials, 45 ET (65 ± 9 years) and 46 controls (64 ± 11 years) were included. Cognitive function and anxiety were assessed using the Montreal Cognitive Assessment (MoCA) and State-Trait Anxiety Inventory (STAI),^30,31^ using validated Chinese versions.^32,33^ ET diagnosis was confirmed by a board-certified neurologist (co-author CH) based on established criteria, including isolated bilateral upper-limb action tremor of ≥1 year duration. Exclusion criteria included other neurological disorders (e.g. parkinsonism, dystonia and cerebellar ataxia), medication- or metabolic-induced tremor and conditions interfering with assessment. Controls were recruited from the community and reported no major neurological or psychiatric history. Groups did not differ in age, sex or trait anxiety, whereas ET showed fewer years of education, lower MoCA scores and higher state anxiety (Table 1). ET patients were stratified a priori by age of onset (<45 versus ≥45 years), family history and response to first-line treatment. Because no prior study has applied the interleaved pro- and anti-saccade paradigm in ET, no ET-specific effect-size estimate was available for an a priori power calculation. We therefore selected a target sample size of 50 participants per group, guided by previous work using this task in healthy and clinical populations^34,35^ and by a power calculation indicating that this sample size provides ∼80% power to detect a moderate-to-large between-group effect of approximately Cohen’s *d* = 0.57 at *α* = 0.05. After trial-quality exclusions, the final analysed sample included 45 ET participants and 46 controls, retaining ∼80% power to detect effects of approximately *d* = 0.59.

**Table 1:** Demographics and clinical scores of participants

Group	Number of participants	Age at time of measurement	Sex (male)	Education (years)	MoCA	STAIS	STAIT
ET	45	64.6 ± 9.5	24	12.1 ± 3.8	23.6 ± 4.6	37.9 ± 10.8	41.6 ± 8.0
CTRL	46	64.0 ± 10.9	19	14.1 ± 3.3	25.6 ± 2.8	19.1 ± 7.4	39.5 ± 8.1
		*P* = 0.77	*P* = 0.25	*P* = 0.011	*P* = 0.014	*P* < 0.001	*P* = 0.23

ET, essential tremor patients; CTRL, healthy age-matched older adults; MoCA, Montreal Cognitive Assessment; STAIS, State-Trait Anxiety Inventory State; STAIT, State-Trait Anxiety Inventory Trait.

### Recording and apparatus

Participants were seated in a dark room with head stabilization. Eye position and pupil size were recorded at 1000 Hz using a video-based eye tracker (EyeLink 1000 Plus; SR Research), with stimulus presentation controlled by Experiment Builder. Stimuli were presented on an LCD monitor (1920 × 1080, 60 Hz) at a viewing distance of 80 cm (43° × 24° visual angle).

### Interleaved pro- and anti-saccade task

The task (Fig. 1A) followed our previous design.^34^ Each trial began with a central fixation point (0.5°, 20 cd/m^2^) on a black background (0.1 cd/m^2^), with colour indicating trial type (pro versus anti; counterbalanced). After 1500 ms, the fixation point disappeared and a peripheral target (0.5°, 270 cd/m^2^) appeared at 8° to the left or right (step paradigm). Participants were instructed to look towards the target (pro-saccade) or to the mirror-opposite location (anti-saccade) as quickly as possible. The task comprised 240 trials in total, divided into two blocks of 120 trials; and each block included 60 pro-saccade and 60 anti-saccade trials. Trial type and target location were randomly interleaved within each block, and left/right responses were collapsed for analysis. Participants received instructions and completed practice trials prior to testing.

**Figure 1:**
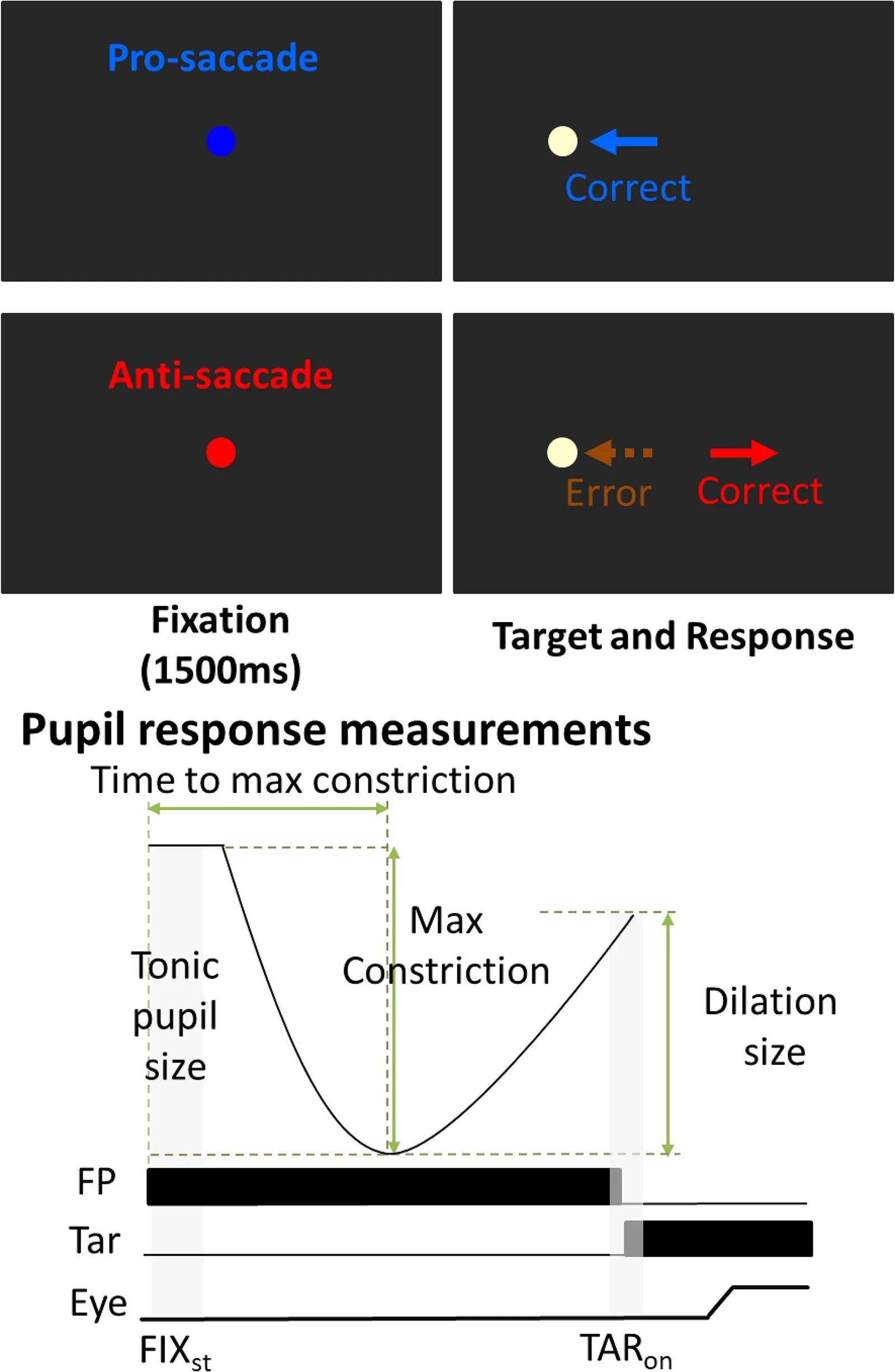
**Task design and pupil response measurements.** In the interleaved pro- and anti-saccade task, participants were instructed to look towards the peripheral target on pro-saccade trials and to suppress the reflexive response and look in the opposite direction on anti-saccade trials. Pupil response measurements included tonic pupil size, maximum constriction, time to maximum constriction and task-evoked dilation size. FP, fixation point; Tar, target; FIX_st, fixation onset; TAR_on, target onset.

### Saccade analysis

Saccade reaction time (SRT) was defined as the interval between target onset and initiation of the first saccade, detected when eye velocity exceeded 30°/s. Trials were classified as correct when the initial saccade was directed towards the target (pro) or the mirror-opposite location (anti), and otherwise as direction errors. Express saccades were defined as short-latency visually triggered saccades within the latency epoch in which erroneous anti-saccades were significantly more frequent than correct anti-saccades. Following established procedures,^34,36^ this epoch was identified using a binomial sign-test across sliding 20 ms windows of the anti-saccade SRT distribution. Windows in which anti-saccade errors exceeded correct anti-saccades at *P* < 0.05 were classified as the express range, corresponding to ∼100–180 ms in the present dataset. Express pro-saccade frequency was then quantified as the proportion of correct pro-saccades falling within this empirically defined latency range. Kinematic measures of the first saccade included amplitude, peak velocity, main sequence slope (peak velocity/amplitude) and endpoint deviation. VOT was derived from the difference between error and correct anti-saccade SRT distributions, smoothed with a seven-point boxcar kernel and defined as the minimum within a 100–500 ms window.^23,37,38^ Trials were excluded if fixation deviated >2° before target onset, kinematic measures exceeded ±3 SD, or endpoint deviation exceeded 8°,^39^ resulting in the exclusion of 9.0% of trials in ET participants and 5.8% of trials in CTRL participants.

### Pupil analysis

Analyses were restricted to central fixation periods prior to saccade initiation and included only correct trials. Pupil data were pre-processed in MATLAB following established procedures,^40^ including detection of invalid samples (e.g. blinks) and linear interpolation. Pupil metrics included one tonic and four phasic indices (Fig. 1). Tonic pupil size was defined as the mean diameter during baseline (0–200 ms after fixation onset) and was subtracted to obtain baseline-corrected measures. Phasic indices included maximum constriction magnitude, time to maximum constriction and pupil size during the target epoch (−50 to 50 ms relative to target onset). Pupil dilation was defined as the difference between target-epoch pupil size and maximum constriction. Pupil and saccadic indices were selected a priori to capture complementary aspects of baseline arousal (tonic pupil size), early autonomic response (maximum constriction) and preparatory modulation (task-evoked dilation), in relation to saccadic initiation (SRT), movement vigour (peak velocity) and spatial accuracy (endpoint deviation), based on prior work linking pupil dynamics to oculomotor control.^34,41^

### Blink analysis

Spontaneous blinks were detected from raw pixel-based pupil signals (1000 Hz). Analyses included only correct trials. Blink samples were defined as low pupil values (<5th percentile), missing samples or velocity outliers (>6 median absolute deviations of the first derivative), with velocity outliers retained only if occurring within ±10 ms of low-value or missing samples. Blink onset impulses were stored as time-by-trial binary matrices. Blink rate dynamics were computed by averaging impulses across trials and converting to blinks per minute. Subject-level blink rate was defined as the mean rate within the preparatory window (−1200 to 0 ms relative to target onset), and blink duration as the mean duration per condition.

### Statistical analysis

Group-level effects were assessed using mixed-design ANOVAs with Group (CTRL versus ET) as a between-subject factor and Task as a within-subject factor. Planned simple effects tested a priori hypotheses, with additional pairwise comparisons conducted using two-tailed independent *t*-tests. Effect sizes are reported as partial eta squared (*η*_p_^2^) and Cohen’s *d*. Because groups differed in education and state anxiety, covariate-adjusted sensitivity analyses were conducted for the main oculomotor and pupillometric outcomes. Repeated-measures outcomes were reanalysed using mixed ANCOVAs with Group as a between-subject factor, Task as a within-subject factor and education years and STAI State (STAIS) scores as covariates. Single-summary outcomes, including VOT and express pro-saccade frequency, were analysed using ANCOVA with the same covariates. These analyses were treated as sensitivity analyses because education and state anxiety may influence task performance and autonomic state. To address potential medication effects, exploratory within-ET analyses compared express pro-saccade frequency across medication groups using Welch ANOVA, with Kruskal–Wallis tests as non-parametric checks. Exploratory associations between MoCA, STAIS and selected anti-saccade measures were assessed using Pearson correlations separately within each group. For trial-by-trial pupil–saccade coupling analyses, subject-level correlation coefficients were Fisher *z*-transformed before group-level testing. Because these analyses involved multiple combinations of pupil measures, saccadic parameters and task conditions, *P*-values were corrected using the Benjamini–Hochberg false discovery rate (FDR) procedure within the pupil–saccade coupling family. Nominal *P-*values are reported descriptively and interpreted cautiously. SRT distributions were compared using a subject-level Kolmogorov–Smirnov (KS) approach, in which each participant’s distribution was tested against the pooled distribution of the opposite group, yielding a single distance measure per subject. Group differences were assessed using Wilcoxon rank-sum tests. Analyses were performed in JASP^42^ and MATLAB.

### Machine learning analysis

To examine whether clinical characteristics of ET were associated with physiological profiles, we implemented a supervised classification framework using repeated nested cross-validation.^43^ Analyses were restricted to the ET group and targeted three binary outcomes: onset <45 years, family history and treatment response. Features were grouped into predefined families (saccade, pupil, blink and cross-domain coupling), and all combinations were evaluated. Within each outer fold, features were z-scored and highly correlated features (|*r*| > 0.90) were removed. Feature selection was performed using univariate area under the receiver operating characteristic curve (AUC) ranking, with top-ranked features retained for model training. Although univariate ranking does not explicitly model higher-order feature interactions, it was used as a conservative dimensionality-reduction strategy given the limited sample size and substantial inter-feature correlations. A ridge-regularized logistic regression classifier was selected to provide stable and interpretable estimation in a small-sample, moderately high-dimensional setting while reducing overfitting risk. The regularization parameter was optimized via inner cross-validation. Performance was quantified using AUC, balanced accuracy, sensitivity and specificity, averaged across repetitions. Statistical significance was assessed using permutation testing.

## Results

### SRT distribution in pro- and anti-saccade task

Figure 2 shows SRT distributions for control (CTRL) and ET groups. Both groups exhibited the characteristic pattern of faster responses in pro-saccades and longer latencies with higher error rates in anti-saccades. Group differences in distributional shape were assessed using subject-level KS distances. No significant group differences were observed for correct pro-saccades (*z* = 0.01, *P* = 0.99) or erroneous anti-saccades (*z* = 1.02, *P* = 0.31). A trend-level difference was observed for correct anti-saccades (*z* = 1.87, *P* = 0.06).

**Figure 2:**
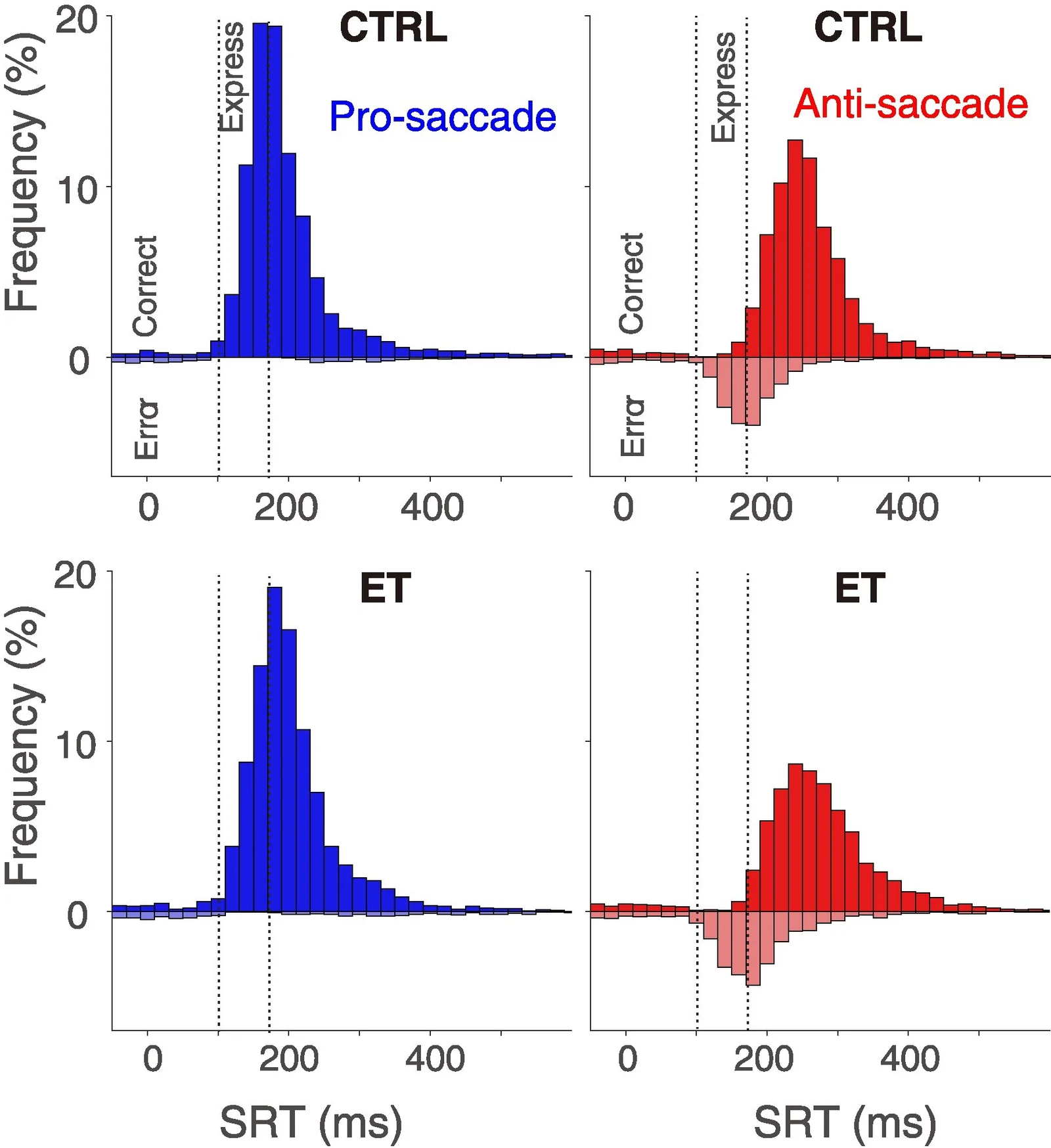
**SRT distributions in pro- and anti-saccade trials.** Distributions of SRTs for pro- and anti-saccades in controls and participants with ET. Dotted vertical lines indicate the short-latency express-saccade range. CTRL, controls, *N* = 46; ET, essential tremor, *N* = 45; SRT, saccade reaction time.

### Saccade latency and metrics

Anti-saccade trials elicited higher direction-error rates than pro-saccades [Fig. 3A, *F*(1,89) = 138.48, *P* < 0.001, *η*_p_^2^ = 0.61], with overall higher error rates in ET compared to CTRL [*F*(1,89) = 4.53, *P* = 0.036, *η*_p_^2^ = 0.048], and no Group by Task interaction (*P* = 0.13). SRTs were longer in anti- than pro-saccades [*F*(1,89) = 422.79, *P* < 0.001, *η*_p_^2^ = 0.83] and were slower overall in ET [*F*(1,89) = 4.82, *P* = 0.031, *η*_p_^2^ = 0.051], again without interaction. Follow-up comparisons indicated that group differences were present for anti-saccades (*P* = 0.020), but not pro-saccades (*P* = 0.15). Because groups differed in education and state anxiety, we conducted covariate-adjusted sensitivity analyses to assess the robustness of the main group effects. In these analyses, controlling for education and STAIS, the group effect on SRT remained significant [*F*(1,86) = 4.55, *P* = 0.036, *η*_p_^2^ = 0.050], including for anti-saccade SRT (*P* = 0.029), whereas the group effect on direction-error rate was attenuated [*F*(1,86) = 1.26, *P* = 0.264, *η*_p_^2^ = 0.015]. Kinematic measures showed robust task effects with minimal group differences. Peak velocity was higher in pro-saccades [*F*(1,89) = 8.35, *P* < 0.01, *η*_p_^2^ = 0.086], whereas amplitude was reduced [*F*(1,89) = 60.33, *P* < 0.001, *η*_p_^2^ = 0.40], resulting in steeper main sequence slopes [*F*(1,89) = 54.01, *P* < 0.001, *η*_p_^2^ = 0.08]. Endpoint accuracy differed by task [*F*(1,89) = 288.64, *P* < 0.001, *η*_p_^2^ = 0.76], with a trend towards greater deviation in ET [*F*(1,89) = 3.85, *P* = 0.053, *η*_p_^2^ = 0.041], and no interactions.

**Figure 3:**
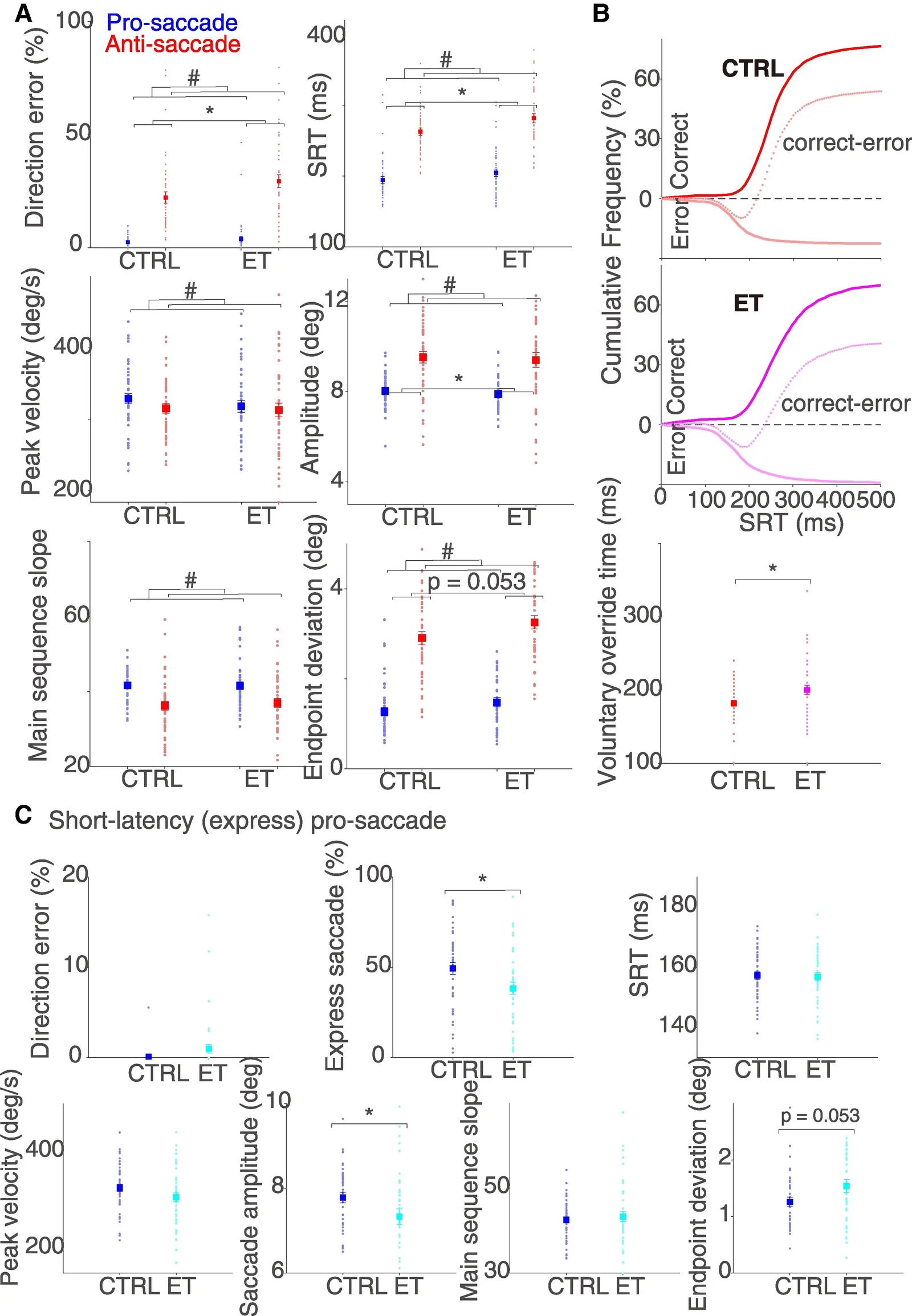
**Oculomotor performance and VOT.** (**A**) Group comparisons of saccadic metrics, including direction-error rate, saccadic reaction time, peak velocity, amplitude, main sequence slope and endpoint deviation. Mixed-design analyses of variance showed group effects for direction-error rate, *F*(1,89) = 4.53, *P* = 0.036, and saccadic reaction time, *F*(1,89) = 4.82, *P* = 0.031; CTRL, *N* = 46; ET, *N* = 45. (**B**) Cumulative saccadic reaction time distributions for correct and erroneous anti-saccades used to derive VOT; CTRL, *N* = 46; ET, *N* = 45. (**C**) Short-latency express pro-saccade analyses, including error rate, express-saccade frequency, saccadic reaction time and kinematic parameters. Group comparisons used two-tailed independent-samples *t*-tests; express-saccade frequency differed between groups, *t*(89) = 2.36, *P* = 0.020; CTRL, *N* = 46; ET, *N* = 45. Data points represent individual participants; error bars indicate mean ± standard error of the mean. *P < 0.05 (group effect); #*P* < 0.05 (task effect). CTRL, controls; ET, essential tremor; SRT, saccade reaction time; VOT, voluntary override time.

### Voluntary override time

Figure 3B shows VOT distributions. VOT was longer in ET than in CTRL [*t*(89) = 2.47, *P* = 0.015, *d* = 0.52]. The VOT difference remained in the same direction after adjustment for education and STAIS but was reduced to trend level [*F*(1,86) = 3.67, *P* = 0.059, *η*_p_^2^ = 0.041]. Exploratory within-group Pearson correlations showed nominal negative associations between MoCA scores and anti-saccade error rate and VOT in both groups, with an additional nominal association with anti-saccade SRT in ET (Supplementary Fig. 1). These analyses were descriptive; *P*-values were not corrected for multiple comparisons and the associations were not interpreted as demonstrating education-independent relationships because MoCA and education are closely related. STAIS scores were not associated with these measures (all *P* > 0.34).

### Short-latency (express) pro-saccades

Figure 3C summarizes short-latency (express) pro-saccades. The proportion of direction errors within the express latency range showed a trend towards being higher in ET than in CTRL [*t*(89) = 1.85, *P* = 0.067, *d* = 0.39]. For correct express saccades, frequency was higher in CTRL than in ET [*t*(89) = 2.36, *P* = 0.02, *d* = 0.50], with no group difference in latency [*t*(89) = 0.23, *P* = 0.82]. The group difference in express pro-saccade frequency remained in the same direction after adjustment for education and STAIS but was attenuated [*F*(1,86) = 2.63, *P* = 0.109, *η*_p_^2^ = 0.030]. In an exploratory within-ET medication analysis, express pro-saccade frequency did not differ significantly across medication groups [no treatment, *n* = 5; β-blocker only, *n* = 9; anti-convulsant only, *n* = 19; combined β-blocker and anti-convulsant treatment, *n* = 8; Welch *F*(3,13.42) = 2.36, *P* = 0.118; Kruskal–Wallis *H* = 4.91, *P* = 0.178], providing no clear evidence that current medication status accounted for reduced express pro-saccade generation. Kinematic measures were largely similar between groups. Peak velocity and main sequence slope did not differ (*P* = 0.096 and *P* = 0.59), whereas ET showed reduced amplitude [*t*(89) = 2.07, *P* = 0.041, *d* = 0.44] and a trend towards greater endpoint deviation [*t*(89) = 1.96, *P* = 0.053, *d* = 0.41].

### Pupil dynamics

Baseline-corrected pupil dynamics during fixation are shown in Fig. 4A, exhibiting an initial constriction followed by dilation in both groups, with task-dependent differences emerging during the preparatory interval. Tonic pupil size did not differ between ET and CTRL [Fig. 4B: *t*(89) = 1.60, *P* = 0.11, Cohen’s *d* = 0.34]. Phasic responses showed clear task modulation. Time to maximum constriction was longer in ET than in CTRL [Fig. 4C: *F*(1,89) = 4.90, *P* = 0.030, *η*_p_^2^ = 0.052], with an additional task effect [longer in pro- than anti-saccades; *F*(1,89) = 21.30, *P* < 0.001] and no interaction (*P* > 0.7). Constriction magnitude differed by task [Fig. 4D: *F*(1,89) = 26.45, *P* < 0.001], without group differences or interaction (*P* > 0.13). Pupil size at target onset was larger in anti- than pro-saccade trials [Fig. 4E: *F*(1,89) = 73.52, *P* < 0.001, *η*_p_^2^ = 0.45], with no group differences or interactions (*P* > 0.18). Preparatory pupil dilation was reduced in ET compared with CTRL [Fig. 4F: *F*(1,89) = 8.07, *P* < 0.01, *η*_p_^2^ = 0.083], and larger in anti- than pro-saccade trials [*F*(1,89) = 35.82, *P* < 0.001, *η*_p_^2^ = 0.29], with no interaction (*P* > 0.9). Education-adjusted analyses showed that the group effect on preparatory pupil dilation remained significant [*F*(1,88) = 6.52, *P* = 0.012, *η*_p_^2^ = 0.069], whereas the group effect on time to maximum constriction was reduced to trend level [*F*(1,88) = 3.56, *P* = 0.062, *η*_p_^2^ = 0.039]. When STAIS was included, group effects on both preparatory pupil dilation and constriction timing were attenuated, either with STAIS alone (*P* = 0.256 and *P* = 0.357, respectively) or together with education (*P* = 0.267 and *P* = 0.376, respectively).

**Figure 4:**
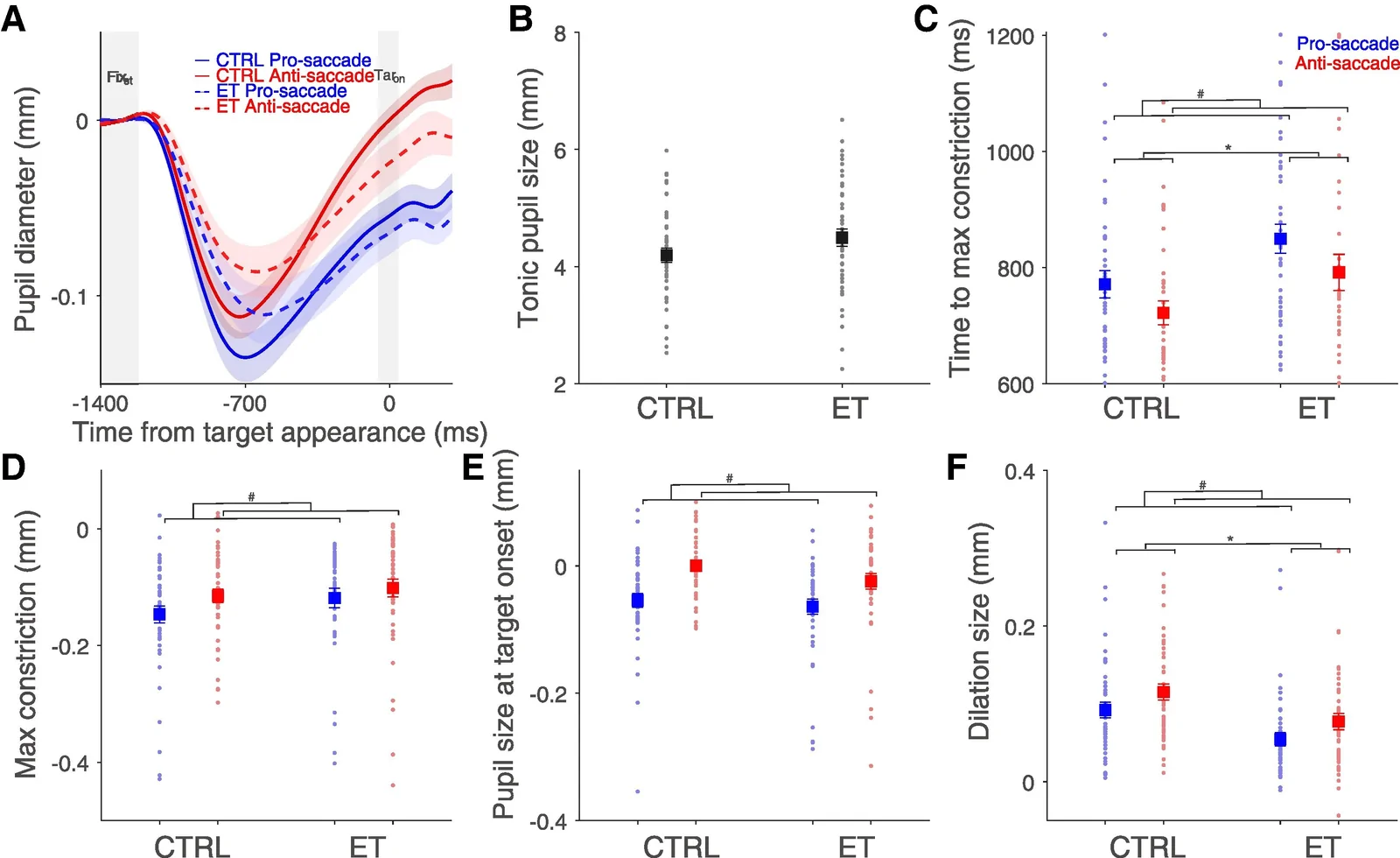
**Pupillary dynamics during the interleaved pro- and anti-saccade task.** (**A**) Time course of pupil diameter aligned to target onset for pro- and anti-saccade trials in controls (CTRL, *N* = 46) and ET participants (ET, *N* = 45). Shaded regions indicate standard error of the mean; grey bars denote fixation and post-target intervals. (**B**) Tonic pupil size across groups, analysed using a two-tailed independent-samples *t*-test [*t*(89) = 1.60, *P* = 0.11]. (**C–F**) Phasic pupil measures were analysed using mixed-design analyses of variance with group and task factors: (**C**) time to maximum pupil constriction [group: *F*(1,89) = 4.90, *P* = 0.030; task: *F*(1,89) = 21.30, *P* < 0.001]; (**D**) maximum constriction amplitude [task: *F*(1,89) = 26.45, *P* < 0.001]; (**E**) baseline-corrected pupil size at target onset [task: *F*(1,89) = 73.52, *P* < 0.001]; (**F**) task-evoked pupil dilation [group: *F*(1,89) = 8.07, *P* < 0.01; task: *F*(1,89) = 35.82, *P* < 0.001]. Data points represent individual participants; error bars indicate mean ± standard error of the mean. *P < 0.05 (group main effect); #*P* < 0.05 (task main effect). CTRL, controls; ET, essential tremor.

### Eye blink dynamics

Eye blink dynamics during the preparatory interval in correct trials are shown in Fig. 5A. Time-resolved blink rate profiles revealed clear task-dependent modulation in both groups, with reduced blinking preceding target onset and a rebound thereafter. Quantitative analysis within the pre-target window (−1200 to 0 ms) demonstrated higher blink rates in the anti-saccade condition compared with the pro-saccade condition [Fig. 5B: *F*(1,89) = 111.67, *P* < 0.001, *η*_p_^2^ = 0.56], with no main effect of group or interaction (*P* > 0.29). Blink duration showed a complementary pattern, with shorter durations in anti- relative to pro-saccade trials [Fig. 5C: *F*(1,89) = 4.57, *P* = 0.035, *η*_p_^2^ = 0.049], again without group differences or interactions (*P* > 0.27).

**Figure 5:**
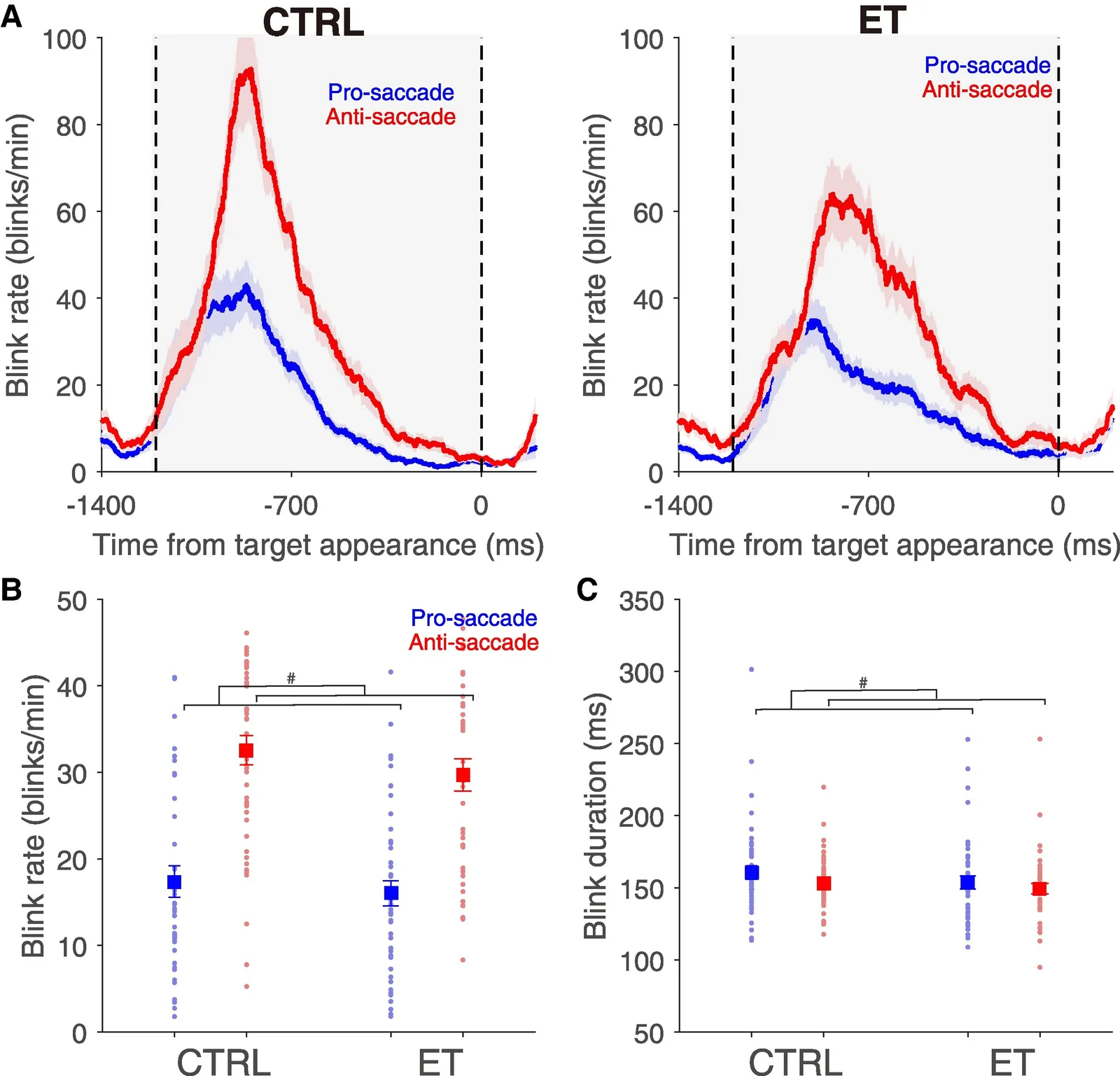
**Blink dynamics during the interleaved pro- and anti-saccade task.** (**A**) Time course of blink rate aligned to target onset for pro- and anti-saccade trials in controls (CTRL, *N* = 46) and ET participants (ET, *N* = 45). Shaded regions indicate standard error of the mean; dashed vertical lines denote analysis windows. (**B–C**) Blink measures were analysed using mixed-design analyses of variance with group and task factors: (**B**) mean blink rate [task: *F*(1,89) = 111.67, *P* < 0.001; group and interaction: *P* > 0.29]; (**C**) blink duration [task: *F*(1,89) = 4.57, *P* = 0.035; group and interaction: *P* > 0.27]. Data points represent individual participants; error bars indicate mean ± standard error of the mean. #*P* < 0.05 (task main effect). CTRL, controls; ET, essential tremor.

### Trial-by-trial coupling between pupil and saccade metrics

Trial-by-trial correlations between pupil measures (tonic size, maximum constriction, dilation) and saccadic parameters (SRT, peak velocity, endpoint deviation) were computed and Fisher *z*-transformed (Fig. 6). Because these analyses involved multiple pupil–saccade combinations, *P*-values were FDR-corrected within the pupil–saccade coupling family. After correction, no between-group differences in coupling strength survived FDR correction. Tonic pupil size showed weak and inconsistent associations with saccadic parameters, with no corrected within-group or between-group effects (Fig. 6A). Maximum constriction also showed no reliable associations with saccadic parameters in either group, and no between-group differences were observed (Fig. 6B). Pupil dilation showed the strongest coupling pattern (Fig. 6C). In particular, pupil dilation was positively associated with peak velocity during pro-saccades in CTRL [*t*(45) = 4.76, *P* < 0.001], and this effect survived FDR correction. The corresponding association in ET was nominally significant [*t*(44) = 2.51, *P* = 0.016], but did not survive FDR correction.

**Figure 6:**
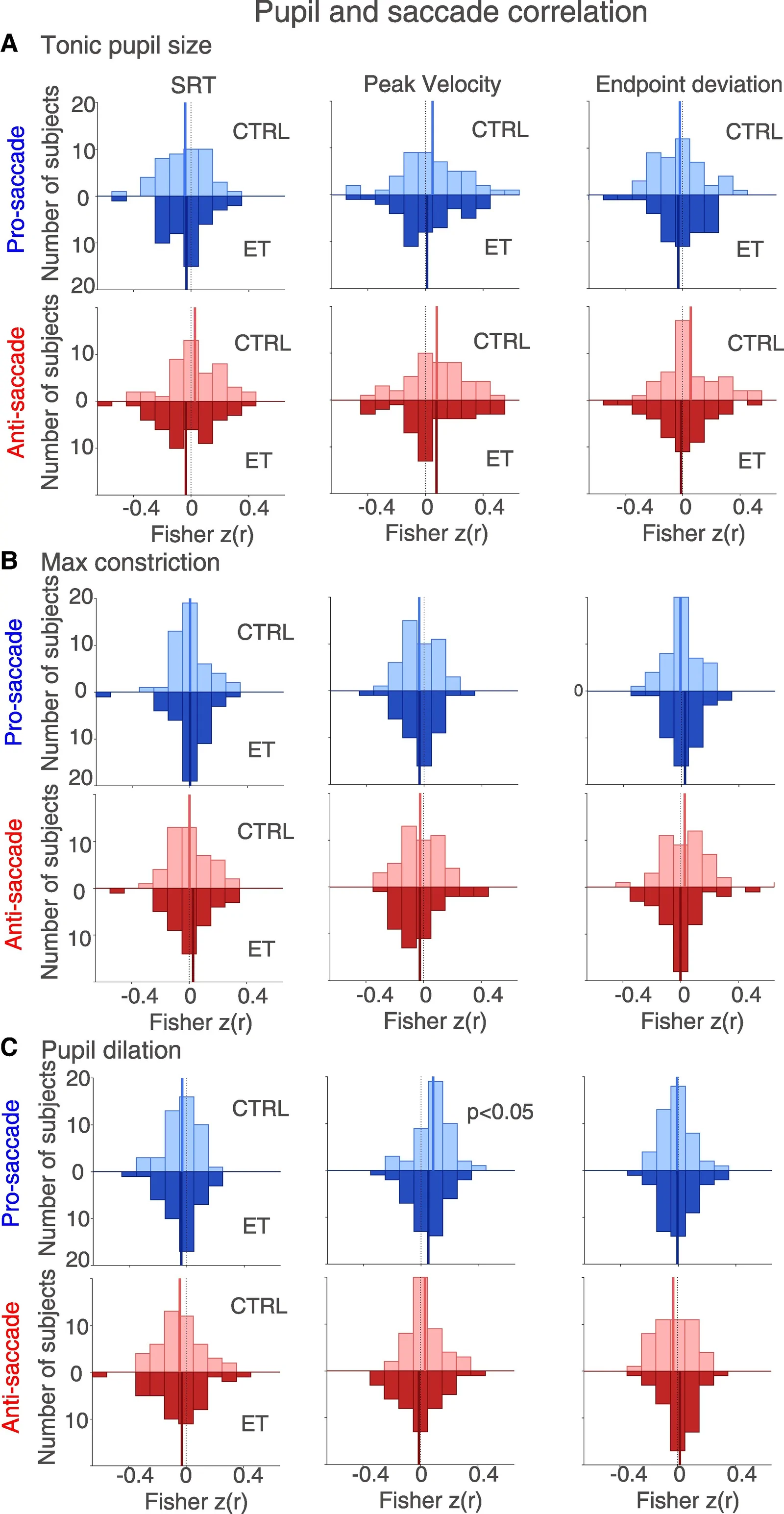
**Trial-by-trial coupling between pupil dynamics and saccadic parameters.** (**A–C**) Distributions of subject-level Fisher *z*-transformed correlation coefficients between pupil metrics and saccadic measures for pro- and anti-saccade conditions in controls (CTRL, *N* = 46) and ET participants (ET, *N* = 45). Pupil metrics include (**A**) tonic pupil size, (**B**) maximum constriction, and (**C**) task-evoked pupil dilation. Saccadic parameters include saccadic reaction time, peak velocity and endpoint deviation. Positive and negative values indicate the direction of within-subject associations. Vertical solid lines indicate zero; dashed lines indicate group means. Significant effects are marked (*P* < 0.05, FDR-corrected). CTRL, controls; ET, essential tremor; SRT, saccade reaction time.

### Blink–pupil coupling

Blink–pupil coupling analyses revealed task-dependent modulation without consistent group differences (Supplementary Fig. 2), indicating that temporal coordination between blink events and pupil dynamics was largely preserved in ET.

### Exploratory machine learning analyses of clinical subtypes

Exploratory machine learning analyses showed modest classification performance across clinical subtypes, with the highest accuracy observed for early onset status using combined pupil–saccade features (AUC = 0.679 ± 0.059), although this did not reach statistical significance (*P* = 0.115). Full results are provided in Supplementary Table 1 and Supplementary Fig. 3.

## Discussion

Using video-based eye tracking during an interleaved pro- and anti-saccade task, we simultaneously characterized oculomotor, pupil and blink dynamics in ET. ET was associated with impaired inhibitory oculomotor control, evidenced by increased direction errors, prolonged anti-saccade latencies, extended VOT and reduced generation of express pro-saccades. In parallel, preparatory pupil responses were attenuated and temporally delayed despite a preserved tonic baseline, consistent with reduced preparatory engagement rather than a global arousal deficit. In contrast, blink dynamics were largely preserved, and most cross-domain physiological relationships remained intact, although blink–pupil coordination showed modest task-dependent effects in raw signals that were not consistently observed after baseline correction. Together, these findings indicate that ET is associated with a distributed but selective physiological disturbance, with prominent abnormalities in oculomotor and autonomic preparatory processes occurring within an otherwise coordinated neural network.

### Oculomotor control and sensorimotor transformation

The oculomotor findings extend prior evidence that ET involves dysfunction within cerebellar and cerebello-thalamo-cortical circuits beyond tremor generation.^4,5^ Increased direction errors, prolonged anti-saccade latencies and extended VOT indicate impaired inhibitory control under increased executive task demands, reflecting reduced efficiency in suppressing or overriding prepotent responses in a context requiring flexible response selection. This pattern aligns with prior reports linking saccade abnormalities in ET to executive dysfunction and disrupted cerebello-thalamo-cortical circuitry.^44^ Mechanistically, these deficits likely arise from dysfunction across partially dissociable cerebellar subcircuits. Elevated anti-saccade error rates and prolonged VOT implicate inefficiency in reciprocal loops linking the lateral cerebellum (dentate nucleus) with dorsolateral prefrontal and frontal eye field regions, supporting top-down suppression of reflexive orienting.^16,25^ In contrast, subtle reductions in saccade amplitude and a trend towards increased endpoint deviation suggest involvement of the medial oculomotor vermis–fastigial nucleus circuit, which supports forward-model-based calibration of saccadic trajectories.^45^ The reduction in express pro-saccades further suggests altered preparatory facilitation of automated visuomotor responses. Express saccades are thought to occur when pre-target preparatory activity in the saccade system is sufficiently elevated such that the transient visual response to target onset rapidly reaches the threshold for saccade initiation.^22,37^ Thus, the lower express pro-saccade frequency in ET may indicate reduced top-down facilitation or altered preparatory set, potentially reflecting disrupted cerebello-thalamo-cortical modulation of fronto-collicular/superior-colliculus mechanisms. Notably, covariate-adjusted analyses indicated that ET-related SRT slowing was relatively robust to education and state anxiety, whereas direction-error, VOT and express-saccade effects were attenuated, suggesting that these measures may partly reflect differences in cognitive reserve or state-dependent task engagement. Together, these findings support a multi-component account in which ET affects inhibitory control under executive task demands, voluntary response efficiency and cerebellar-dependent spatial calibration as partially dissociable but co-occurring impairments, rather than reflecting a unitary inhibitory deficit.

### Pupillometric dynamics and autonomic modulation

Pupillometric results extend this interpretation to pupil-linked autonomic and neuromodulatory modulation. ET participants showed reduced task-evoked pupil dilation with preserved tonic pupil diameter, a mechanistically informative dissociation. Within the adaptive gain framework,^17^ pupil diameter has been used as an indirect marker of LC–NE-related arousal state, with tonic diameter linked to baseline arousal and task-evoked dilation linked to phasic responses to task demands. The selective attenuation of task-evoked dilation in ET, with preserved task sensitivity, suggests reduced preparatory gain in pupil-linked arousal signals rather than impaired task engagement or global arousal changes. This interpretation is broadly consistent with emerging evidence implicating brainstem and LC-related systems in ET, including reports of brainstem pathology and neuromelanin-sensitive MRI markers associated with cognitive status.^46-48^ Functionally, pupil dynamics are shaped by interactions among cortical, subcortical and brainstem systems, including pathways involving the SC, which integrates arousal, sensory and cognitive signals during orienting.^49,50^ Reduced preparatory dilation in ET may therefore reflect altered coordination within this broader network, rather than a deficit localized to a single pathway. The delayed time to maximum constriction likely reflects altered responses between early light-evoked constriction and later task-related dilation, rather than a primary deficit in the constriction mechanism itself, as constriction magnitude was preserved. Consistent with this interpretation, prior work has reported subtype-dependent alterations in reflexive and feedback-related pupil responses in ET.^51,52^ However, because the group effects on pupil dilation and constriction timing were attenuated after STAIS adjustment, these pupillometric differences may reflect ET-related changes in preparatory autonomic modulation that partly overlap with state-anxiety-related arousal. Together, these findings suggest that task-evoked autonomic modulation is sensitive to ET-related physiological change, while also being influenced by state-dependent autonomic factors.

### Blink dynamics

In contrast to oculomotor and pupillary measures, blink dynamics were largely preserved at the group level.^20^ Both groups showed comparable spontaneous blink rates and task-dependent modulation, including reduced blink rate preceding target onset and shorter blink durations during anti-saccade preparation. This preservation of task-related blink suppression suggests that mechanisms supporting blink inhibition under top-down cognitive demand remain largely intact, arguing against a global disruption of brainstem motor control. Notably, prior work has demonstrated impaired eyeblink conditioning in ET, reflecting dysfunction of olivo-cerebellar circuits even in early disease stages.^53^ This dissociation suggests that conditioning deficits may primarily reflect impaired predictive motor learning, whereas spontaneous, task-related blink suppression depends on partially distinct neural mechanisms.^21,54^ The coexistence of preserved spontaneous blink dynamics and impaired conditioning is therefore mechanistically coherent, indicating that olivo-cerebellar pathology in ET may selectively disrupt learned timing of motor outputs without broadly affecting blink generation. Finally, potential effects of β-blocker medication on blink rate via adrenergic modulation of orbicularis oculi activity cannot be excluded and may have partially obscured subtle group differences.

### Cross-domain physiological coordination

Cross-domain analyses did not reveal FDR-corrected group differences at the trial-by-trial level, indicating no robust evidence that pupil–saccade coupling was altered in ET. Among pupil components, task-evoked dilation showed the most consistent nominal associations with saccadic behaviour, particularly with peak velocity and, to a lesser extent, SRT, whereas tonic pupil size and constriction-related effects were comparatively weak. Although most individual associations did not survive correction for multiple comparisons, this descriptive pattern is broadly consistent with prior work linking pupil dilation to saccadic preparation and execution.^34,41^ It is also consistent with the proposed role of integrative midbrain circuits, including the SC, in coordinating arousal-related and oculomotor processes.^49,55^ Within this framework, the absence of corrected group differences suggests that pupil–saccade coordination may be broadly preserved in ET, despite reduced preparatory pupil dilation. Thus, ET may involve reduced gain of pupil-linked preparatory arousal signals without clear evidence of disrupted trial-by-trial cross-domain coupling. Consistent with this interpretation, modest task-dependent blink–pupil effects further suggest attenuated autonomic responsiveness without a global disruption of cross-domain physiological coordination.

### Cross-domain markers of clinical heterogeneity

The supplementary machine-learning analyses were exploratory and should be interpreted cautiously. Although the highest numerical performance was observed for early onset status using pupil–saccade coupling features, this result was not externally validated and did not survive permutation testing. Therefore, the apparent domain-specific patterns should be interpreted as hypothesis-generating rather than as clinically deployable subtype classifiers. They may suggest candidate cross-domain eye-tracking features for future studies of ET heterogeneity, but should not be taken as evidence for a specific LC–NE mechanism. Classification of family history and medication response remained modest and near chance, further emphasizing the preliminary nature of these analyses. Larger, prospectively designed cohorts with external validation will be required to determine whether pupil–saccade features reliably capture clinically meaningful ET subtypes.

### Limitations

The interleaved pro- and anti-saccade paradigm should not be interpreted as an isolated measure of oculomotor inhibition. Because trial types were randomly intermixed, performance required attentional monitoring, working-memory maintenance of task rules and flexible response selection. Accordingly, the observed oculomotor abnormalities in ET should be interpreted as impaired inhibitory control under executive task demands, rather than as a deficit in an isolated inhibitory mechanism. Although speed–accuracy trade-offs may contribute to performance, the coexistence of slower anti-saccade responses and elevated direction-error rates argues against a simple compensatory slowing account. In addition, because the task uses highly constrained stimulus–response rules, these findings should be interpreted as laboratory-based indices of oculomotor control rather than direct measures of naturalistic visuomotor behaviour. Although the sample size was substantial for this methodology, power to detect subtle group differences in cross-domain coupling and clinical subgroup analyses remains limited. ET participants had lower education and higher state anxiety than controls. Covariate-adjusted analyses indicated that ET-related SRT slowing was relatively robust, whereas direction-error rate, VOT and express-saccade frequency were attenuated after adjustment for education and STAIS. In contrast, pupillometric effects were particularly sensitive to STAIS adjustment, suggesting partial overlap with state-anxiety-related autonomic state. Medication effects could not be fully resolved because medication subgroups were small and treatment status was not randomized. Exploratory within-ET analyses did not show a significant medication-group difference in express pro-saccade frequency. However, medication effects on other physiological measures cannot be excluded; for example, β-blockers may influence blink rate via adrenergic mechanisms and pupil size via sympathetic modulation, potentially obscuring group differences in these measures. Moreover, while the pupillometric results are consistent with altered pupil-linked arousal modulation, they provide only indirect evidence regarding LC–NE-related mechanisms. The absence of concurrent neuroimaging therefore limits direct inference regarding structural LC involvement. In addition, the supplementary machine-learning analyses were exploratory, not externally validated, and did not survive permutation testing; the conservative linear pipeline may also have missed non-linear or higher-order multivariate interactions. These findings should therefore be considered hypothesis-generating and require validation in larger, prospectively designed cohorts. Finally, the cross-sectional design precludes conclusions about disease-stage dependence of the observed physiological profiles.

## Conclusion

ET is associated with impaired inhibitory oculomotor control and selectively attenuated preparatory pupil dilation, consistent with dysfunction in cerebello-thalamo-cortical circuits and altered autonomic–arousal regulation. Blink dynamics and cross-domain coupling remain largely preserved, indicating selective rather than global disruption of sensorimotor and autonomic processes. Notably, patterns of pupil–saccade coupling showed sensitivity to clinical heterogeneity, particularly early onset status, although these findings were exploratory and require validation. Multimodal eye tracking therefore provides a scalable, non-invasive window into this distributed pathophysiology and offers a physiological framework for characterizing the heterogeneous expression of ET.

## Supplementary Material

fcag389_Supplementary_Data

## Data Availability

Due to privacy and consent restrictions, individual-level data are not publicly available but can be shared in anonymized form upon request to the corresponding author, subject to institutional approvals. MATLAB scripts used in the data analysis employed standard functions and packages that are freely available; no new package or function was generated for this study.
