# Supplementary material for: Multimodal eye-tracking reveals impaired inhibitory control and reduced preparatory pupil dilation in essential tremor
Source: Brain Commun. 2026 Oct 15;8(5):fcag389. doi: 10.1093/braincomms/fcag389 (PMC13714541; doi:10.1093/braincomms/fcag389)
Supplement: Supplementary file 1 [file fcag389_supplementary_data.pdf]

Supplementary Table 1. **Performance of machine-learning models for classification of essential tremor clinical subtypes.** Exploratory machine-learning analyses were restricted to ET participants with available clinical subtype information. Models were trained using repeated nested cross-validation across predefined feature families and their combinations. Reported values represent mean ± SD across folds for area under the curve (AUC), balanced accuracy, sensitivity, and specificity. Feature families include saccade, pupil, blink, and cross-domain coupling features. Classification targets included early-onset status (onset <45 years), family history, and medication response. Overall performance was modest, and permutation testing did not indicate significance above chance levels. Values are mean ± SD across repeated nested cross-validation. Class balance is shown as class0/class1. Only nonredundant metrics used in the manuscript are reported.

Target	Feature family	Class balance	No. features	AUC	Balanced accuracy	Sensitivity	Specificity
Onset <45	All features	30/13	63	0.563 ± 0.072	0.631 ± 0.056	0.546 ± 0.200	0.717 ± 0.203
Onset <45	Saccade only	30/13	20	0.467 ± 0.038	0.572 ± 0.030	0.462 ± 0.299	0.683 ± 0.323
Onset <45	Pupil only	30/13	9	0.384 ± 0.067	0.541 ± 0.023	0.508 ± 0.438	0.573 ± 0.427
Onset <45	Blink only	30/13	4	0.468 ± 0.076	0.570 ± 0.052	0.723 ± 0.215	0.417 ± 0.259
Onset <45	Pupil-saccade corr only	30/13	18	0.668 ± 0.046	0.713 ± 0.042	0.815 ± 0.150	0.610 ± 0.108
Onset <45	Blink-pupil dynamics only	30/13	12	0.404 ± 0.049	0.545 ± 0.021	0.869 ± 0.259	0.220 ± 0.240
Family History	All features	24/19	63	0.498 ± 0.062	0.578 ± 0.045	0.526 ± 0.336	0.629 ± 0.347
Family History	Saccade only	24/19	20	0.602 ± 0.040	0.649 ± 0.047	0.732 ± 0.135	0.567 ± 0.074
Family History	Pupil only	24/19	9	0.612 ± 0.043	0.645 ± 0.024	0.747 ± 0.232	0.542 ± 0.228
Family History	Blink only	24/19	4	0.388 ± 0.070	0.535 ± 0.031	0.632 ± 0.368	0.438 ± 0.359
Family History	Pupil-saccade corr only	24/19	18	0.497 ± 0.055	0.570 ± 0.036	0.553 ± 0.334	0.588 ± 0.321
Family History	Blink-pupil dynamics only	24/19	12	0.400 ± 0.059	0.521 ± 0.028	0.421 ± 0.379	0.621 ± 0.348
Response to 1st medi	All features	20/23	63	0.384 ± 0.052	0.523 ± 0.028	0.496 ± 0.301	0.550 ± 0.301
Response to 1st medi	Saccade only	20/23	20	0.323 ± 0.048	0.516 ± 0.031	0.326 ± 0.438	0.705 ± 0.419
Response to 1st medi	Pupil only	20/23	9	0.535 ± 0.068	0.607 ± 0.051	0.439 ± 0.178	0.775 ± 0.170
Response to 1st medi	Blink only	20/23	4	0.388 ± 0.053	0.510 ± 0.038	0.204 ± 0.298	0.815 ± 0.263
Response to 1st medi	Pupil-saccade corr only	20/23	18	0.457 ± 0.076	0.561 ± 0.047	0.387 ± 0.300	0.735 ± 0.258
Response to 1st medi	Blink-pupil dynamics only	20/23	12	0.430 ± 0.063	0.545 ± 0.032	0.635 ± 0.372	0.455 ± 0.377

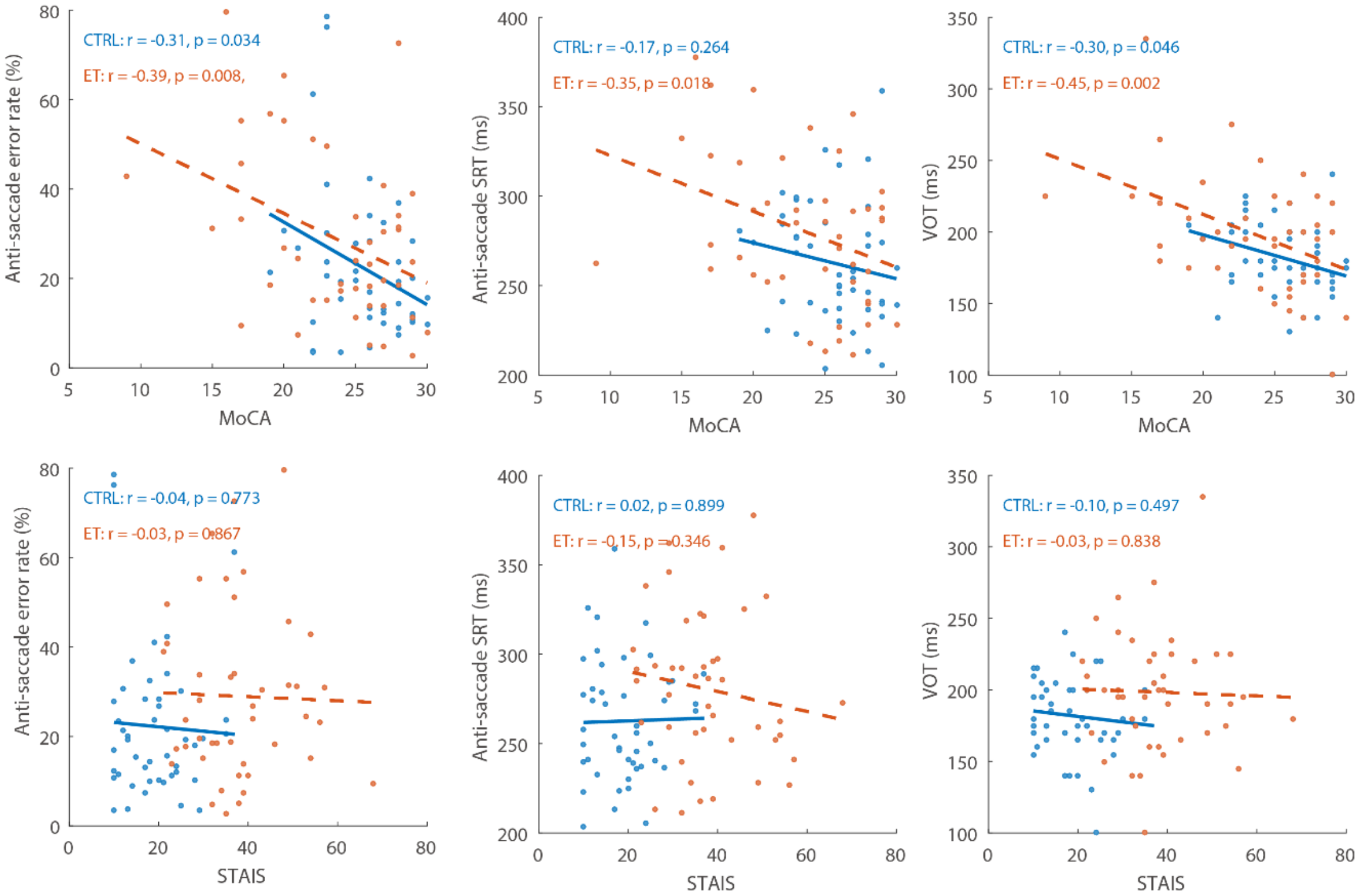

Supplementary Figure 1. Associations between cognitive performance (MoCA) and state anxiety (STAIS) with saccadic measures in essential tremor (ET) and controls (CTRL). Exploratory within-group Pearson correlations are shown for anti-saccade error rate, anti-saccade SRT, and VOT. MoCA scores showed nominal negative associations with anti-saccade error rate and VOT in both groups, with an additional nominal association with anti-saccade SRT in ET. STAIS scores were not associated with these measures. Each datapoint represents one participant. P-values are uncorrected and should be interpreted descriptively. MoCA: Montreal Cognitive Assessment; STAIS: State–Trait Anxiety Inventory State.

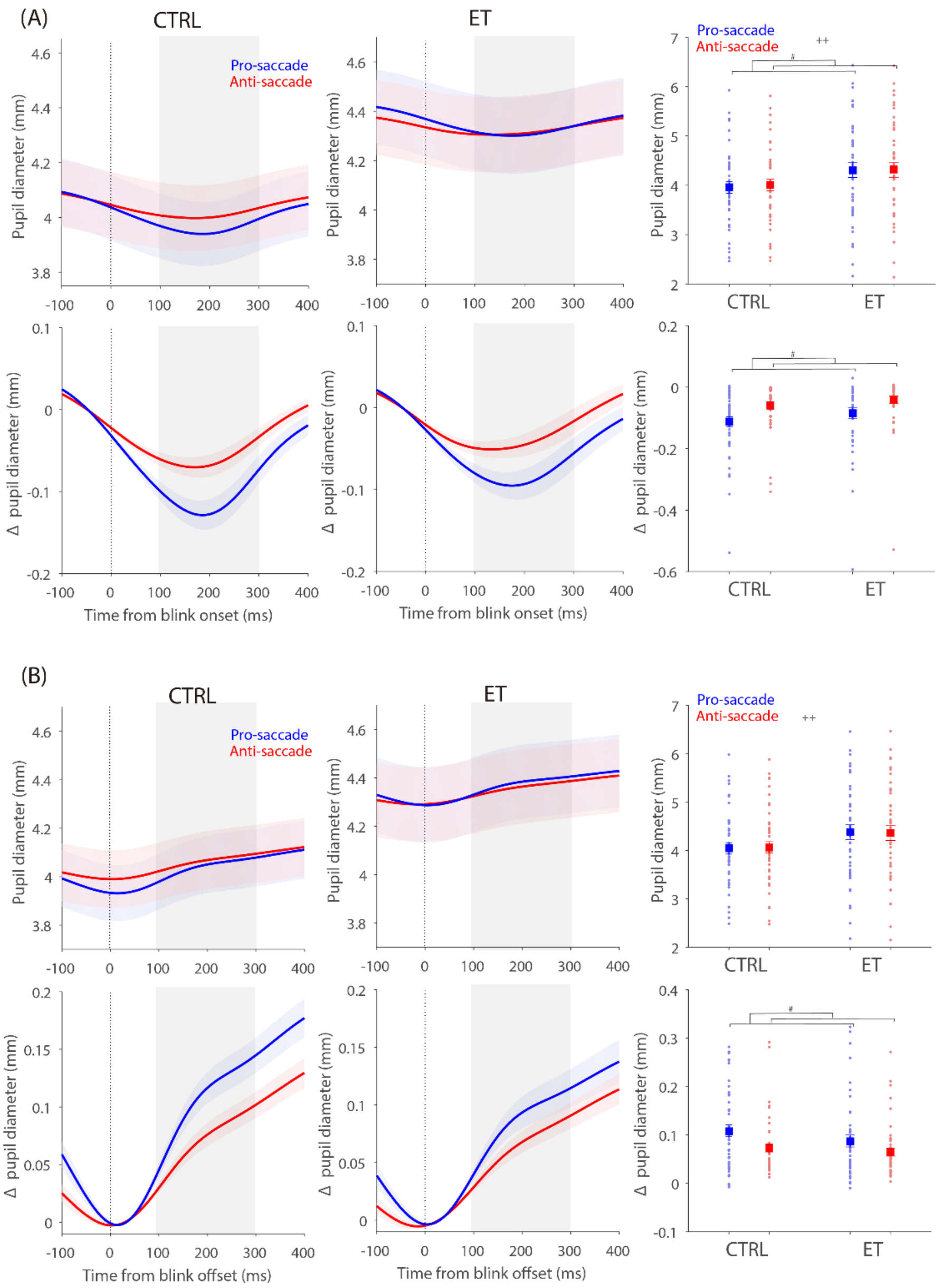

Supplementary Figure 2. Temporal coupling between blink events and pupil dynamics. Analyses included controls (CTRL, N = 46) and essential tremor participants (ET, N = 45), and responses were analysed using mixed-design analyses of variance with group and task factors. (A) Pupil diameter and baseline-corrected responses aligned to blink onset for pro- and anti-saccade trials in controls and essential tremor participants. A transient pupil constriction was observed following blink onset. Responses were analysed using mixed-design analyses of variance with group and task factors. In the raw signal, there was a main effect of task ($F(1,89) = 5.66$, $P = 0.019$, $\eta p^2 = 0.060$) and a group by task interaction ($F(1,89) = 4.54$, $P = 0.036$, $\eta p^2 = 0.049$), with no main effect of group ($P = 0.088$). In the baseline-corrected signal, constriction was larger in pro- than anti-saccade trials ($F(1,89) = 53.70$, $P < 0.001$, $\eta p^2 = 0.376$), with no group effect or interaction ($P > 0.22$). (B) Pupil diameter and baseline-corrected responses aligned to blink offset. A rebound dilation was observed

following blink offset. In the raw signal, no main effects of task or group were found ($P > 0.10$), but an interaction was present ($F(1,89) = 4.17$, $P = 0.044$, $\eta p^2 = 0.045$). In the baseline-corrected signal, a main effect of task was observed ($F(1,89) = 27.66$, $P < 0.001$, $\eta p^2 = 0.237$), with no group effect or interaction ($P > 0.22$). Shaded regions indicate standard error of the mean; gray shaded areas denote analysis windows; dashed lines indicate blink onset or offset. Right panels show mean responses within the analysis window. Data points represent individual participants; error bars indicate mean ± standard error of the mean. *$P < 0.05$ (group main effect); ++$P < 0.05$ (interaction). CTRL: controls; ET: essential tremor.

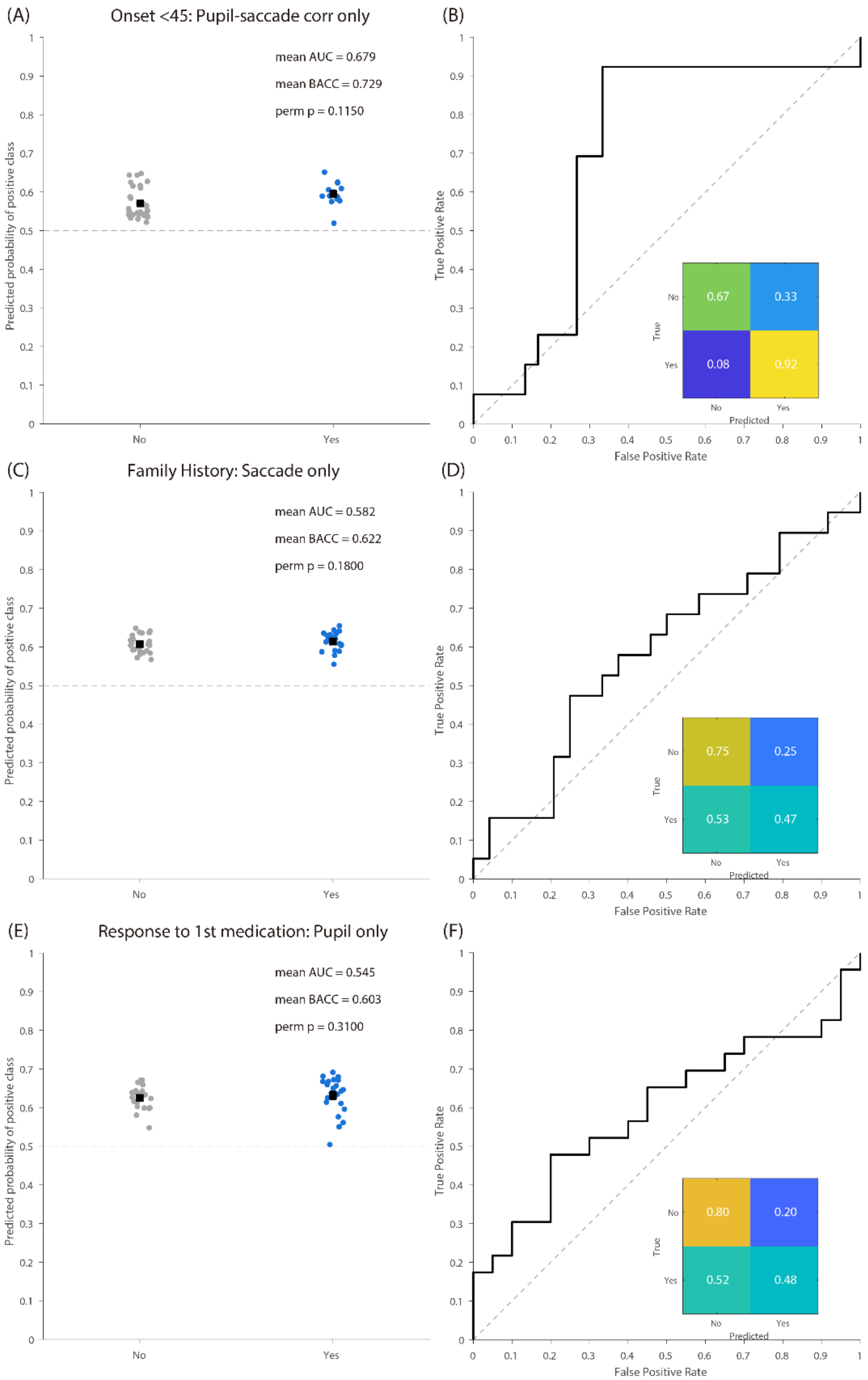

Supplementary Figure 3. Exploratory machine learning classification of clinical subtypes in essential tremor. (A, C, E) Predicted probabilities of class membership for individual participants based on models using (A) pupil–saccade coupling features for early-onset status (onset <45 years; class balance, 30/13; N = 43), (C) saccade features for family history (class balance, 24/19; N = 43), and (E) pupil features for response to first-line medication (class balance, 20/23; N = 43). Each datapoint represents one participant's cross-validated predicted probability; black squares indicate group means. (B, D, F) Receiver operating characteristic

curves for the corresponding models, with insets showing normalized confusion matrices and dashed diagonal lines indicating chance-level performance; N = 43 for each analysis. For early-onset status, classification performance was highest using pupil–saccade features (A, B; area under the receiver operating characteristic curve = 0.679 ± 0.059; balanced accuracy = 0.729 ± 0.047; sensitivity = 0.841; specificity = 0.617), although this did not reach statistical significance (permutation-test P = 0.115). Classification of family history (C, D; area under the receiver operating characteristic curve = 0.582; balanced accuracy = 0.622; permutation-test P = 0.18) and medication response (E, F; area under the receiver operating characteristic curve = 0.545; balanced accuracy = 0.603; permutation-test P = 0.31) was modest and near chance. AUC: area under the receiver operating characteristic curve; BACC: balanced accuracy; perm p: permutation-test P-value.
